# Improving Access to the Glycated Hemoglobin Test in Rural Communities With Point-of-Care Devices: An Application Study

**DOI:** 10.3389/fmed.2021.734306

**Published:** 2021-11-22

**Authors:** Marianne Silveira Camargo, Luiz Carlos Santana Passos, Sostenes Mistro, Daniela Arruda Soares, Clavdia Nickolaevna Kochergin, Vivian Carla Honorato dos Santos de Carvalho, Jéssica Caline Lemos Macedo, Taciana Borges Andrade Cortes, Amós Alves de Souza, Davi Rumel, Marcio Galvão Oliveira

**Affiliations:** ^1^Program of Post-graduation in Medicine and Health, Federal University of Bahia, Salvador, Brazil; ^2^Program of Post-graduation in Collective Health, Multidisciplinary Institute of Health, Federal University of Bahia, Vitória da Conquista, Brazil; ^3^Multidisciplinary Institute of Health, Federal University of Bahia, Vitória da Conquista, Brazil; ^4^Department of Community Health, School of Medicine, Municipal University of São Caetano do sul, São Caetano Do Sul, Brazil

**Keywords:** diabetes mellitus, glycated hemoglobin A, point-of-care testing, primary health care, rural communities

## Abstract

**Background:** Living in a rural or remote area is frequently associated with impaired access to health services, which directly affects the possibility of early diagnosis and appropriate monitoring of diseases, mainly non-communicable ones, because of their asymptomatic onset and evolution. Point-of-care devices have emerged as useful technologies for improving access to several laboratory tests closely patients' beds or homes, which makes it possible to eliminate the distance barrier.

**Objective:** To evaluate the application of point-of-care technology for glycated hemoglobin (HbA1c) estimation in the assessment of glycemic control and identification of new diagnoses of diabetes in primary care among rural communities in a Brazilian municipality.

**Materials and Methods:** We included individuals aged 18 years or older among rural communities in a Brazilian municipality. From September 2019 to February 2020, participants were assessed for anthropometrics, blood pressure, and capillary glycemia during routine primary care team activities at health fairs and in patient groups. Participants previously diagnosed with diabetes but without recent HbA1c test results or those without a previous diagnosis but with random capillary glycemia higher than 140 mg/dL were considered positive and were tested for HbA1c by using a point-of-care device.

**Results:** At the end of the study, 913 individuals were accessed. Of these, 600 (65.7%) had no previous diagnosis of diabetes, 58/600 (9.7%) refused capillary glycemia screening and 542/600 (90.7%) were tested. Among tested individuals, 73/542 (13.5%) cases without a previous diagnosis of diabetes, were positive for capillary glycemia. Among positives, 31/73 (42.5%) had HbA1c levels that were considered indicative of prediabetes and 16/73 (21.9%) were newly diagnosed with diabetes. Among the participants, 313/913 (34.3%) were previously diagnosed with diabetes. Recent HbA1c results were unavailable for 210/313 (67.1%). These individuals were tested using point-of-care devices. Among them, 143/210 (68.1%) had HbA1c levels higher than target levels (>7% and >8% for adults and elderly individuals, respectively.

**Conclusion:** The application of point-of-care devices for HbA1c level measurement improved the access to this test for people living in rural or remote areas. Thus, it was possible to include this technology in the routine activities of primary health care teams, which increased the rates of new diagnoses and identification of patients with uncontrolled glycemia.

## Introduction

Diabetes mellitus (DM) affects more than 460 million adults worldwide, of whom an estimated 152.6 million individuals live in rural or remote areas (1). Such patients face greater difficulty in properly managing their condition because of the difficulty in accessing medical care and a lack of regular monitoring of glycated hemoglobin (HbA1c) levels and therapeutic drugs (2). Among the most recent technologies developed for the care of patients with DM, point-of-care devices for the estimation of HbA1c levels have been shown to have the potential to improve access to regular monitoring of HbA1c levels for people with difficulty in accessing health services (3, 4). Further, people who live far from urban centers are at a significantly higher risk of not having their health problems properly diagnosed or monitored (5). This risk is directly correlated with the distance from the urban center of reference (2) and is often associated with the need for traveling long distances to access health services without the availability of a viable means of transportation or by bad access routes (3, 6, 7). Consequently, the greater the distance from urban centers, the greater is the susceptibility to complications related to chronic health conditions or early death (2).

According to the last demographic census of Brazil, 15.6% of the population lives in rural or remote areas (8). There are almost 16.8 million people with DM in the country, with an estimated prevalence of 11.4% (9). The mortality rate due to diabetes in Brazil is estimated to be around 30.7/100 thousand inhabitants, and approximately 50% of individuals with DM are undiagnosed (9). The care of people living with DM, as well as other chronic non-communicable diseases (NCDs), is the responsibility of primary care teams, which are overburdened in most municipalities. Often, these teams have to deal with a high prevalence of health conditions with little technological support apparatus (10). These limitations reduce the quality of care and make it more difficult to obtain more satisfactory control rates in patients with DM and other NCDs, compared to that richer in countries (11).

HbA1c levels are considered the best predictor of chronic complications in patients with DM, reflect the severity of glycemia in the last 3–4 months, and have low variability. Furthermore, fasting is not required for the estimation of these levels (12–14). Despite the advantages of HbA1c estimation, the need for precision and standardization of analytic methods limits its application outside urban centers (15, 16). In addition, in the Brazilian scenario, the results of the test performed in laboratories are not immediately available, which may mean an even greater delay in reviewing the treatment of a patient with uncontrolled glycemia or in confirming a new diagnosis. On the other hand, the use of point-of-care devices to measure HbA1c has been shown to be cost-effective in the care of DM patients in primary care, with the possibility of improving glycemic control rates in a timely manner to reduce the development of DM-related complications, and consequently, their economic impact on health systems (17). Therefore, this study aimed to evaluate the application of point-of-care technology for HbA1c estimation in the assessment of glycemic control and identification of new diagnoses of diabetes in primary care among rural communities in a Brazilian municipality.

## Materials and Methods

This application study was conducted with a cross-sectional analysis. The analysis included individuals aged 18 years or older residing in rural areas covered by family health units with or without a previous diagnosis of diabetes from September 2019 to February 2020.

### Study Location

The municipality where the study was conducted is in northeastern Brazil and has a total area of 3,254,186 km^2^. Its estimated population is 338,480 inhabitants, of which approximately 12% live in rural areas (18). The rural area selected has 18 primary health care units, with 100% coverage of the rural population through the family health program. The municipality has a highly complex public laboratory that performs most laboratory tests for the patients attended to via the local public health system. The farthest rural unit is 82 km away from the municipal laboratory.

**Figure 1:**
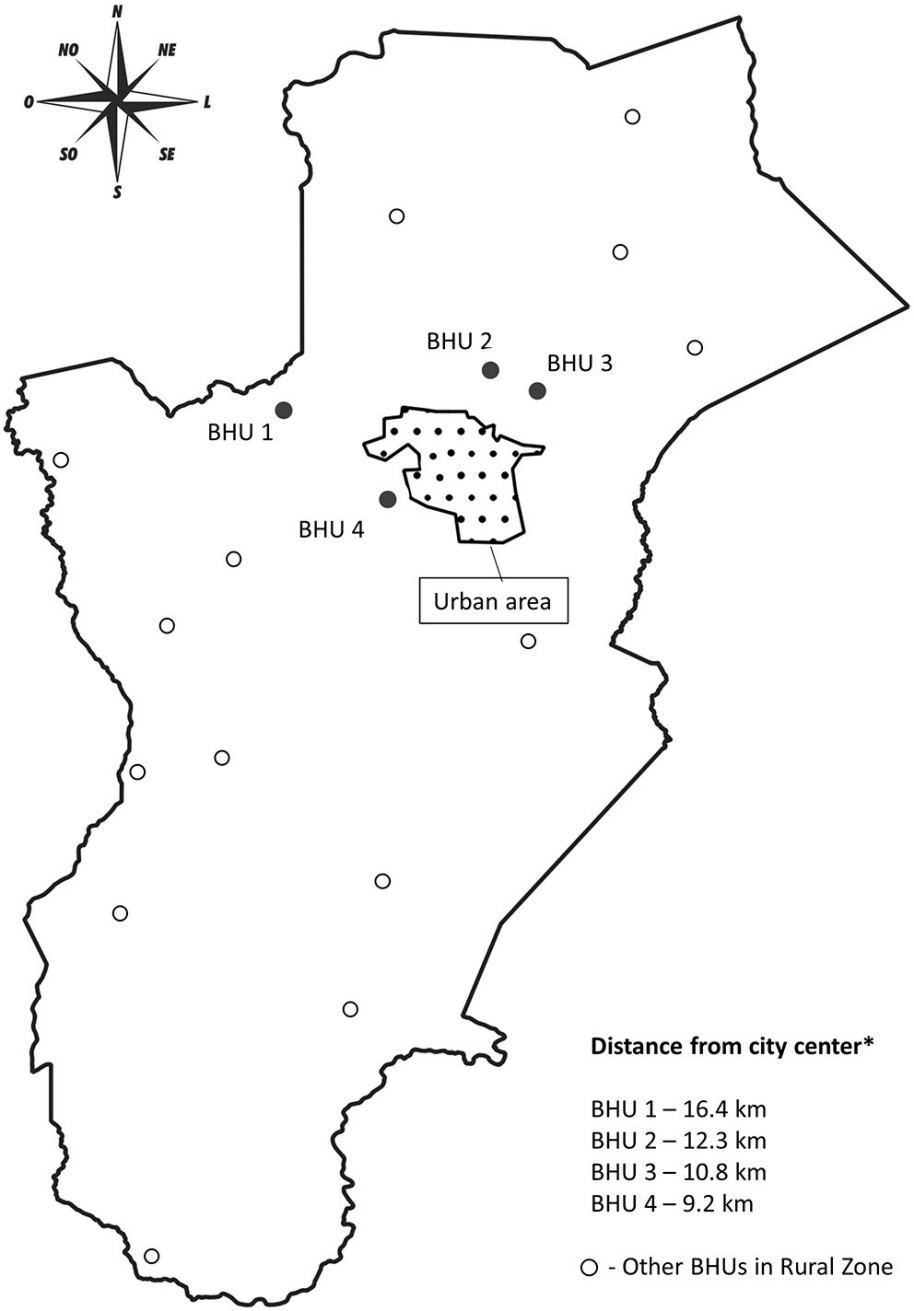
Map of the municipality with the geospatial distribution of the basic health units in the rural area of study.

Four health units in the rural area were included in the study because of their proximity to the urban area, in view that it would be easy for the teams in these units to perform the necessary actions. Each unit has a team comprising a nurse, a dentist, a doctor, nursing technicians, and community health workers (CHWs). The basic health units where the study was conducted were chosen by convenience, considering the number of patients attended to by each of them, referral of patients for exams at the central laboratory, and the engagement of health care teams for participation in the project. Figure 1 shows the map of the municipality with the geographic distribution of all health units in the rural area, the delimitation of the urban area, and the location of the municipal laboratory. The units included in the study were named BHU 1 to 4.

### Provision of HbA1c Tests

HbA1c measurements were performed in two ways. Initially, health fairs were conducted in the rural family health team's coverage area, where the study was conducted. After this stage, the equipment was taken to the corresponding BHU on pre-scheduled dates on which patient groups were attended to at the units.

#### Health Fairs

The first stage consisted of four health fairs, one in each area covered by the BHU included in the study, with the participation of the research team and teams from the participating units themselves. The invitation to the health fairs was extended by CHWs through home visits. Only individuals with hypertension and/or diabetes living in the areas covered by each unit were invited. However, access to and participation in the fairs were not restricted to this population. The individuals participated in health education activities and answered a questionnaire aimed at collecting demographic and socioeconomic data. Anthropometric, blood pressure, and capillary blood glucose measurements were performed. Participants without a previous diagnosis of diabetes with random capillary blood glucose levels ≥ 140 mg/dL, and those with a previous diagnosis of diabetes but for whom HbA1c test results in the last 3 months were unavailable, underwent HbA1c level measurement using the Abbott Afinion ™ 2 Analyzer. This device was chosen based on two criteria: 1. it was registered in Brazil by the national regulatory agency; 2. validation studies results of the device were available (19). Patients with a previous diagnosis of diabetes and HbA1c levels higher than the control target (>7% and >8% for adults and the elderly, respectively) and those without a previous diagnosis of diabetes and HbA1c levels of ≥ 6.5% were referred to the attending physician in at the primary care units. For the present study, a new diagnosis of diabetes was defined as no prior diagnosis of diabetes and an HbA1c level of ≥ 6.5%. After the initial evaluation at health fairs, individuals who tested positive or with HbA1c level higher than the control target were asked to repeat the test within 3 months at the primary care unit. The follow-up of these patients has been described in another study (20).

#### Patient Groups

Similar to health fairs, patient group activities are part of the routine of family health teams. These activities involve educational initiatives and assessments conducted by nurses and physicians, such as blood pressure, capillary glycemia, and anthropometry measurements. Participants with measurements higher than the corresponding threshold values were attended to by the physician at the unit. During the study period, a portable device for the measurement of HbA1c was provided at the BHU for the days on which activities were scheduled for patient groups with diabetes or hypertension, particularly for those with an indication to repeat the HbA1c test, identified at health fairs. The criteria for HbA1c level evaluation were the same as those applied at health fairs and included individuals for whom a new measurement was indicated. Due to the COVID-19 pandemic, subsequent measurements were suspended from March 2020.

### Ethical Aspects

The study was approved by the Ethics Committee on Research in Human Beings of the Multidisciplinary Institute in Health–Campus Anísio Teixeira (Opinion number: 3.357.963). The requirement of patient's informed consent was waived because the study could not be conducted without a waiver and involved no more than minimal risk.

## Results

**Figure 2:**
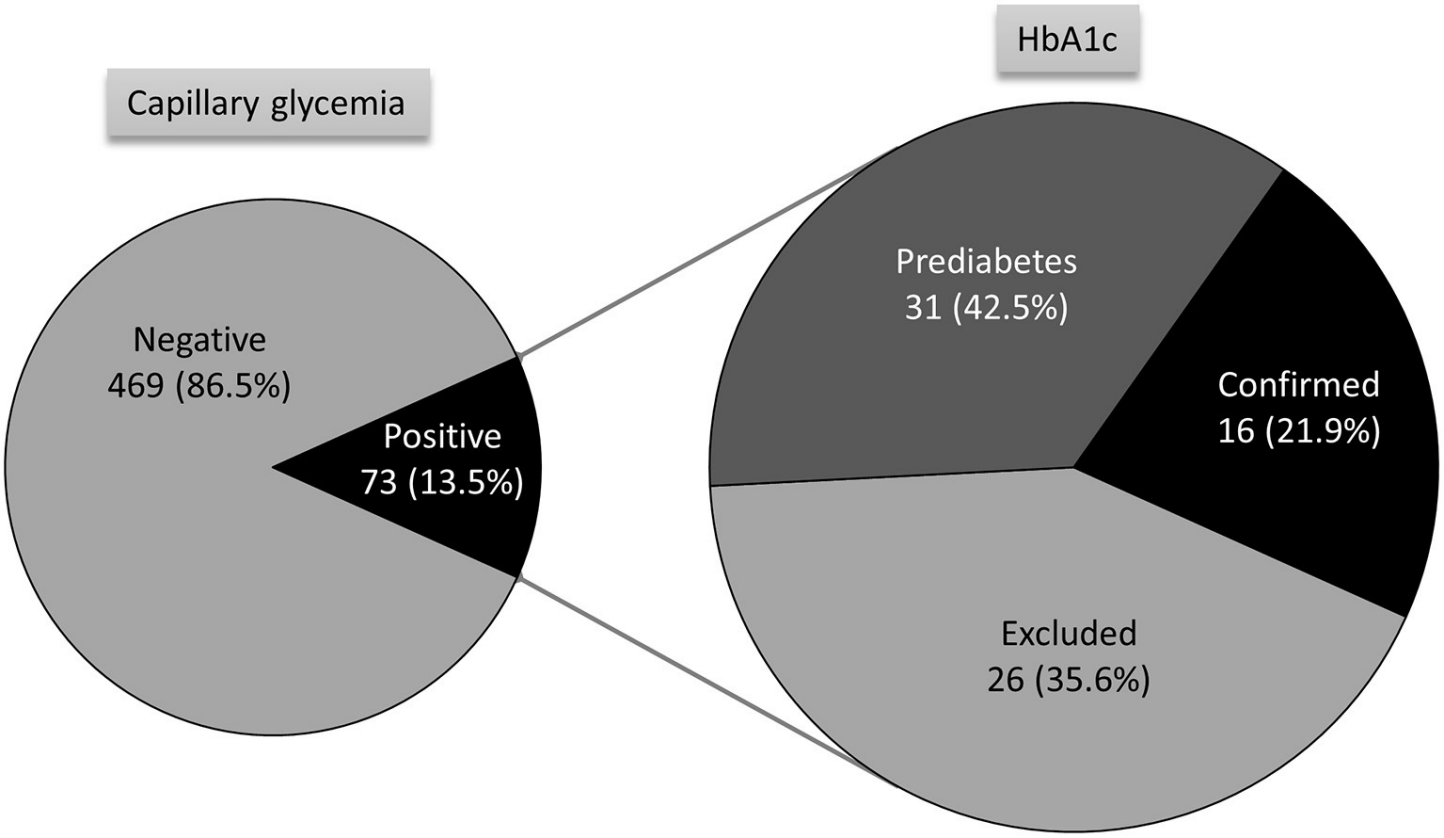
Results of the screening for capillary glycemia and diagnosis confirmation by HbA1c testing performed using point-of-care test devices.

During this period, 913 individuals were observed. Table 1 describes the main characteristics of the participants in terms of their health status. Among the measurements performed for the evaluation of control and diagnosis, 283 measurements of HbA1c level were performed. Of the total participants, 600 individuals had no previous diagnosis of diabetes. Among these individuals, 542 (90.3%) were included in the screening by capillary blood glucose measurement and 58 (9.7%) refused to take the test. Figure 2 shows the results of screening for diabetes and HbA1c measurements via point-of-care performed in individuals without a previous diagnosis of diabetes but capillary blood glucose levels higher than the control threshold. Among the participants with a previous diagnosis of diabetes, 210 (67.1%) had no recent HbA1c results available and were tested using a point-of-care device to assess glycemic control. Of the patients evaluated, 143 (68.1%) had HbA1c levels higher than the control target. Table 2 shows the main characteristics of the individuals whose HbA1c levels were within and exceeded the control target, according to the HbA1c threshold for each age group.

**Table 1:** Main characteristics of the participants who visited the health fairs and patient groups.

**Characteristic**	***n***	**%**
**Sex**		
Female	606	66.4
Male	307	33.6
**Age group (years)**		
<40	61	6.7
40–59	358	39.2
60–79	425	46.5
≥80	69	7.6
**Ethnicity (self-reported)**		
White	179	19.6
Brown	454	49.7
Black	234	25.6
Other	46	5.0
**Literacy status**		
Literate	582	63.7
Illiterate	331	36.3
**Basic health unit**		
BHU 1	210	23.0
BHU 2	275	30.1
BHU 3	197	21.6
BHU 4	231	25.3
Previous diagnosis of hypertension	834	91.3
Previous diagnosis of diabetes	313	34.4
**Average monthly income (US$)**		
Family		303.10
Personal		117.66

**Table 2:** Comparison of the characteristics of the participants with a previous diagnosis of diabetes according to the glycemic status assessed using the HbA1c point-of-care test.

	**Controlled**		**Uncontrolled**		**Prevalence ratio (95% CI)**	***p*-value**
	***n***	**%**	***n***	**%**		
**Age group**						
Adult	18	15.7%	97	84.3%	1	< 0.001
Older people	49	51.6%	46	48.4%	1.742 (1.395–2.175)	
**Sex**						
Female	42	30.0%	98	70.0%	1	0.402
Male	25	35.7%	45	64.3%	1.089 (0.887–1.337)	
**Diagnosis of hypertension**						
No	10	26.3%	28	73.7%	1	0.414
Yes	57	24.9%	172	75.1%	1.102 (0.887–1.369)	
**Capilary glycemia**						
Normal	25	78.1%	7	21.9%	1	0.000
High	40	32.0%	85	68.0%	0.322 (0.165–0.814)	
**BMI**						
Normal	11	28.2%	28	71.8%	1	0.615
Overweight/Obese	30	24.2%	94	75.8%	0.947 (0.760–1.181)	
**Ethnicity**						
White	14	31.8%	30	68.2%	1	
Brown	21	26.3%	59	73.8%	1.049 (0.819–1.342)	0.706
Black	12	26.7%	33	73.3%	1.041 (0.788–1.376)	0.776
**Physically active**						
No	47	34.8%	88	65.2%	1	0.363
Yes	16	28.1%	41	71.9%	0.906 (0.739–1.111)	
**Average monthly income (US$)**						
Family	300.63		319.45		30.86 (-42.48–80.13)	0.543
Personal	112.72		119.08		12.44 (-18.33–31.10)	0.609

## Discussion

In the present study, we demonstrated the application of point-of-care devices for the measurement of HbA1c levels in the detection of new cases and monitoring of glycemic control in patients with diabetes in four rural areas in a Brazilian municipality. The equipment was included in routine activities of the primary health care teams, such as health fairs and group visits. In 6 months, 16 new cases of diabetes and 31 individuals with prediabetes were identified. Furthermore, 210 had no HbA1c results in the past 3 months, of which 68.1% had HbA1c levels higher than the control threshold. Although the areas we studied are covered by the family health program, with primary care units located in these locations, the efforts of the health teams have not been sufficient to identify individuals with diabetes, those with high potential for the development of diabetes, or those with inadequately controlled blood glucose levels.

Several factors, such as socioeconomic status, can make it difficult to perform tests to screen for new cases and adequately monitor chronic NCDs, which results in failures in the management of these conditions (21). Among the factors that influence care in the case of NCDs as well as care in remote locations (5), in our study, the distance from the urban center seemed to directly affect the access to health services. This impression is reinforced when comparing data of a recent study conducted in the urban area of the same municipality, with a similar screening methodology, in which the rate of new diagnoses of diabetes among the individuals screened was 1.2% (22). On the other hand, in our study, this rate was 13.5%. In addition to the difference in the overall rate of new cases of diabetes identified between urban and rural areas, there was a significant difference in the probability of detecting new cases of diabetes between the locality further from the city center and those closer to it in the four rural areas. This finding corroborates the data of the 9 report which estimated that 46% of people living with diabetes in Brazil were undiagnosed (9).

Although the highest rates of glycemic control have been observed in the elderly population, more than half of the participants in the health initiatives were older than 60 years, with some being older than 80 years of age. This portion of the population is the most vulnerable when individuals are required to travel great distances to access health services. Therefore, ensuring these technologies are available closer to the place of residence for such individuals could reduce the need for travel and afford greater access to these services and a better quality of life (21).

The higher prevalence of HbA1c level exceeding the control threshold was associated with the adult age group (age of < 65 years). In addition to living outside the urban center, this group of individuals may face other barriers to accessing health services, such as the working hours of the primary care units in the localities or of the public laboratory for collecting blood samples for the measurement of HbA1c. These services generally operate on working days and during business hours, which makes it impossible for the rural population to participate in these activities during these periods because it interferes with their work. Thus, the availability of point-of-care devices in routine health actions, performed on alternative days such as weekends, can greatly benefit these people and, possibly, is one of the causes of the highest rate of non-control in this group.

The main international guidelines for the management of diabetes as well as the Brazilian guidelines, define the need for at least two confirmatory tests to confirm the diagnosis of diabetes. However, we used a single measure of HbA1c, after a random capillary blood glucose level ≥ 140 mg/dL, to define new cases. This decision was based on the low probability that an individual with hyperglycemia, associated with HbA1c levels that reflect the average blood glucose levels in the last 3 months, is a false positive for diabetes. However, we present the possibility of overestimating the newly diagnosed patients in the studied population and highlight that these patients were referred for diagnostic confirmation to the physicians at the health units in their localities.

Primary care attributes include health education, screening and management of NCDs, and referral of patients to specialized care (23). In the present study, some of the cases considered as those of new diagnoses of diabetes had an HbA1c level > 8%. It is expected that these individuals had already presented symptoms of hyperglycemia, such as thirst and changes in diuresis. However, they had not yet been diagnosed with the condition. There may have been several direct causes for this finding, such as the neglect of symptoms and the consequent inertia in seeking health care, the absence of screening for diabetes in the localities, or even a delay in conducting diagnostic examinations. All these possibilities converge to a root cause: the difficulty in accessing health services and the consequent failure in the early detection of diabetes.

The main consequences of failure in glycemic control and delay in the detection of diabetes are micro-and macrovascular lesions, which result in cardiovascular and renal complications, loss of functional capacity, and early mortality (24). These complications, in addition to significantly affecting the quality of life and survival of people with diabetes, have a high financial impact on health systems. In Brazil, the available data have attributed an average annual cost with complications related to diabetes between 112.90 and 3917.00 dollars per patient/patient/year, which depends on the type of complication presented, resulting from inadequate control (17). When considering these values, the estimated cost for the local health system with complications related to diabetes ranges from 17,047.90 to 591,467.00 dollars, only for patients from these four locations whose HbA1c levels were higher than the control threshold.

Our results suggest the need to expand the access of diabetes patients for regular monitoring of HbA1c levels in rural areas of the municipality. How this access can be expanded depends on the financial and logistical capacity of the local public health system. In this study, the implementation of the use of point-of-care devices proved to be a feasible alternative, due to its practicality in the places where health care services were conducted by primary care teams and in which there was a large participation of the rural population. The initiative made it possible to measure HbA1c in people who needed it in these areas. In addition, we recently demonstrated that this type of technology can be cost-effective if applied in primary care units in urban areas (17). Given the findings obtained with the application of point-of-care for HbA1c measurement in rural communities, we believe that the cost-effectiveness ratio of the initiative is even more favorable when compared to testing in laboratories.

Our study has some limitations. First, we highlight that providing HbA1c testing to diagnose and monitor diabetes will not necessarily correspond to health improvements. However, in a previous study on the same population, although the reduction in the mean HbA1c was not significant, we observed a reduction of HbA1c by one percentage point in 38% of the included patients, which suggests a significant reduction in their cardiovascular risk and probably reflects revised pharmacotherapy and reinforcement of the efforts of the healthcare team, combined with life style changes adopted by the patients. Unfortunately, the follow-up of these patients was interrupted due to the COVID-19 pandemic, and most patients were only examined once during the follow-up. Moreover, our results may be overestimated because of a potentially biased population sample, as it may be possible that only patients with a relatively low self-perceived health status sought out the health services. However, as discussed above, health fairs and patient groups are part of the routine activities of primary care teams in Brazil, and our study was designed to assess the application of the HbA1c-POC technology in a real-world scenario. Therefore, we believe the participants of this study represent, as closely as possible, the population these rural areas.

In conclusion, in this study, we demonstrated that the use of point-of-care devices to measure HbA1c can increase access for people living in rural or remote areas. It was possible to include the technology in the routine activities of primary health care teams, which led to new diagnoses and identification of many patients with an uncontrolled glycemic status in a health system that had failed to regularly monitor HbA1c levels.

## Data Availability Statement

The raw data supporting the conclusions of this article will be made available by the authors, without undue reservation.

## Ethics Statement

The studies involving human participants were reviewed and approved by Ethics Committee on Research in Human Beings of the Multidisciplinary Institute in Health–Campus Anísio Teixeira. Written informed consent for participation was not required for this study in accordance with the national legislation and the institutional requirements.

## Author Contributions

MO, SM, DS, CK, and VC contributed to the study conception and design. Data acquisition, analysis and interpretation, and the manuscript draft were performed by MC, MO, SM, JM, TC, and AS. DR and LP actively contributed to the critical revision of the manuscript. All authors contributed to the article and approved the submitted version.

## Funding

This work was funded by the Medtronic Foundation under Grant Number 255790 from CAF America. This study was financed in part by the Coordenação de Aperfeiçoamento de Pessoal de Nível Superior–Brasil (CAPES)–Finance Code 001.

## Conflict of Interest

The authors declare that the research was conducted in the absence of any commercial or financial relationships that could be construed as a potential conflict of interest.

## Publisher's Note

All claims expressed in this article are solely those of the authors and do not necessarily represent those of their affiliated organizations, or those of the publisher, the editors and the reviewers. Any product that may be evaluated in this article, or claim that may be made by its manufacturer, is not guaranteed or endorsed by the publisher.
